# Stability and survival analysis of elderly patients with osteolytic spinal bone metastases after palliative radiotherapy: Results from a large multicenter cohort

**DOI:** 10.1007/s00066-019-01482-1

**Published:** 2019-06-25

**Authors:** Tilman Bostel, Robert Förster, Ingmar Schlampp, Tanja Sprave, Sati Akbaba, Daniel Wollschläger, Jürgen Debus, Arnulf Mayer, Heinz Schmidberger, Harald Rief, Nils Henrik Nicolay

**Affiliations:** 1 Department of Radiation Oncology, University Hospital of Heidelberg, Im Neuenheimer Feld 400, 69120 Heidelberg, Germany; 2 Clinical Cooperation Unit Radiation Oncology, German Cancer Research Center (DKFZ), Im Neuenheimer Feld 280, 69120 Heidelberg, Germany; 3 Department of Radiation Oncology, University Medical Center Mainz, Langenbeckstraße 1, 55131 Mainz, Germany; 4 Department of Radiation Oncology, University Hospital of Zurich, Raemistraße 100, 8091 Zurich, Switzerland; 5 Department of Radiation Oncology, University Hospital of Freiburg, Robert-Koch-Straße 3, 79106 Freiburg, Germany; 6 Institute of Medical Biostatistics, Epidemiology and Informatics (IMBEI), University Medical Center Mainz, Mainz, Germany

**Keywords:** Neoplasm metastases, Spine, Geriatrics, Fracture, Aged, Metastasen, Wirbelsäule, Geriatrie, Fraktur, Ältere Patienten

## Abstract

**Purpose:**

This retrospective study aimed to evaluate the stability and fracture rates of osteolytic spinal bone metastases (SBM) in elderly patients following palliative radiotherapy (RT) and to derive prognostic factors for stability and survival.

**Methods:**

A total of 322 patients aged at least 70 years received palliative RT at two major German academic medical centers or at the German Cancer Research Center. Stability assessment was based on the validated Taneichi score prior to RT and at 3 and 6 months after RT. The survival time following RT was assessed, and prognostic factors for stability and survival were analyzed.

**Results:**

Prior to RT, 183 patients (57%) exhibited unstable SBM and 68 patients (21%) pathological fractures. At 3 and 6 months after RT, significant recalcification and stabilization were evident in 19% (23/118) and 40% (31/78) of surviving patients, respectively. Only 17 patients (5%) experienced new pathological fractures following RT. Tumor histology was found to significantly influence stabilization rates with only breast cancer patients exhibiting increased stabilization compared to patients with other histologies. The median survival time and 6‑month survival rates following RT were 5.4 months (95% confidence interval 4.4–7.2 months) and 48%, respectively. The patients’ performance status was found to be the strongest predictor for survival after RT in this patient cohort; further factors demonstrating a significant association with survival were the application of systemic treatment, the number of SBM and the primary tumor histology. To analyze the influence of age on survival after RT, study patients were stratified into 3 age groups (i.e., 70–74 years, 75–79 years, and ≥80 years). The subgroup of patients aged at least 80 years showed a strong trend towards a worse survival time following RT compared to younger patients (i.e., 6‑month survival rate 39% vs. 51%; *p* = 0.06, log-rank test).

**Conclusions:**

Prognostic factors influencing overall survival such as performance status and histology should guide the choice for palliative RT for SBM. Strongly hypofractionated RT regimes may be advisable for most elderly patients considering the overall poor prognosis in order to reduce hospitalization times.

## Zusammenfassung

**Hintergrund:**

In dieser retrospektiven Analyse wurden osteolytische spinale Knochenmetastasen von geriatrischen Patienten nach palliativer Radiotherapie (RT) hinsichtlich der Stabilisierungs- und Frakturraten untersucht, zudem wurden prognostische Faktoren für Stabilität und Überleben analysiert.

**Methoden:**

Insgesamt 322 Patienten mit einem Alter von mindestens 70 Jahren wurden an zwei deutschen Universitätskliniken oder am Deutschen Krebsforschungszentrum (DKFZ) mit einer palliativen RT behandelt und im Rahmen der Studie analysiert. Die Stabilität wurde anhand des validierten Taneichi-Scores vor RT sowie 3 und 6 Monate nach RT beurteilt. Die Überlebenszeit nach RT wurde erfasst und prognostische Faktoren bezüglich Stabilität und Überleben analysiert.

**Ergebnisse:**

Vor RT wiesen 183 Patienten (57%) instabile Knochenmetastasen und 68 Patienten (21%) pathologische Frakturen auf. Nach 3 und 6 Monaten zeigten sich bei 19% (23/118) und 40% (31/78) der überlebenden Patienten eine signifikante Rekalzifikation und Stabilisierung. Neue pathologische Frakturen wurden nach RT bei 17 Patienten (5%) nachgewiesen. Die Tumorhistologie hatte signifikanten Einfluss auf die Stabilisierungsraten, wobei Patienten mit Mammakarzinom höhere Stabilisierungsraten als Patienten mit anderen Tumorentitäten erzielten. Das mediane Überleben und die 6‑Monats-Überlebensrate nach RT betrugen 5,4 Monate (95%-Konfidenzintervall 4,4–7,2 Monate) und 48%. Der stärkste Prädiktor für das Überleben nach RT war der Allgemeinzustand (AZ) der Patienten; weitere Faktoren, die einen signifikanten Zusammenhang mit dem Überleben nach RT zeigten, waren die Verabreichung einer Systemtherapie, die Anzahl der Knochenmetastasen und der histologische Befund des Primärtumors. Um den Einfluss des Alters auf die Überlebenszeit nach RT zu analysieren, erfolgte eine Stratifikation der Studienpatienten in 3 Altersgruppen (70–74 Jahre, 75–79 Jahre und ≥80 Jahre). Dabei zeigte sich für die Patienten mit einem Alter von mindestens 80 Jahren ein starker Trend hin zu einer schlechteren Überlebenszeit nach RT als bei jüngeren Patienten (6-Monats-Überlebensrate 39% vs. 51%; *p* = 0,06 Log-Rang-Test).

**Schlussfolgerungen:**

Prognostische Faktoren für das Überleben, wie der AZ und die Tumorhistologie, sollten bei der Wahl der palliativen RT von instabilen Knochenmetastasen beachtet werden. In Anbetracht der meist schlechten Prognose empfiehlt sich für die meisten geriatrischen Patienten die Anwendung stark hypofraktionierter Bestrahlungsprotokolle, um unnötige Hospitalisierungszeiten zu vermeiden.

## Background

Improvements in general health care and oncologic advances have resulted in strongly increasing numbers of geriatric cancer patients [1]. However, due to restrictions in the performance status, prevalent comorbidities or poor treatment tolerance, general therapy guidelines often do not apply to these elderly patients, requiring dedicated trials [2].

Spinal bone metastases (SBM) from solid tumors affect a considerable number of patients and are of particular concern for geriatric patients, as they may more easily result in immobilization and dependence on full-time care [3]. Additionally, relevant comorbidities such as osteoporosis are more prevalent in the elderly and may increase the risk of SBM-related impairments such as pathological fractures [4].

Palliative radiotherapy (RT) constitutes a mainstay in the treatment of SBM and aims at reducing pain and supporting recalcification, resulting in improved bone stability [5, 6]. RT dose and fractionation are commonly adjusted depending on the extent of metastatic disease, comorbidities, performance status, and estimated life expectancy [7–13]. The influence of palliative RT on the analgesic effects and improvements in neurological deficits have already been subject to previous investigations [9, 10, 12]. However, the benefit of RT for the recalcification and stabilization of initially unstable SBM in geriatric patients is still largely unknown. A deeper understanding of these treatment effects of RT will be relevant to choose appropriate treatment algorithms with regard to treatment time and dose.

Therefore, this retrospective study aimed to assess osteolytic SBM in a large multicenter cohort of elderly patients over 70 years regarding stability, fracture rates before and after RT, survival, and prognostic factors for stability and survival.

## Patients and methods

### Patient selection

The medical records of 322 elderly patients with SBM treated with palliative RT at the University Hospitals of Heidelberg and Mainz and the German Cancer Research Center were retrospectively assessed. Patient data from cancer registries of participating centers were collected to include patients aged 70 years or older receiving palliative RT for SBM between March 2000 and January 2014. The diagnosis of SBM was based on imaging procedures such as computed tomography (CT), magnetic resonance imaging (MRI) or bone scintigraphy. Inclusion criteria of this analysis were an age ≥70 years, an osteolytic phenotype of SBM and location of metastases in the thoracic or lumbar spine.

### Stability assessment

**Fig. 1:**
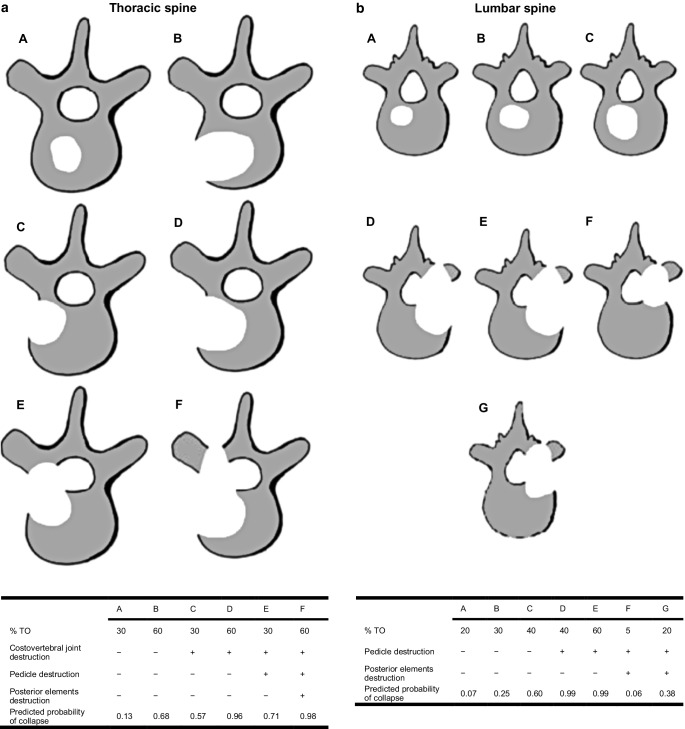
**a** Taneichi score of the thoracic spine, **b** Taneichi score of the lumbar spine *TO* Percentage of metastatic bone destruction in the vertebral body. (Modified from [19])

To assess the stability of osteolytic SBM, the validated Taneichi bone stability score was based on the planning CT scans and two consecutive follow-up CT scans conducted at 3 months (follow-up 1) and 6 months (follow-up 2) after RT. The applied scoring system is based on purely radiological criteria and is therefore a simple method for classifying osteolytic SBM in stable and unstable lesions [14]. For all patients included in this dataset, stability assessments were performed by a board-certified radiologist: Osteolytic SBM were rated on a scale from A to G based on radiological risk factors such as the degree of vertebral body tumor affectation, costovertebral joint or pedicle destruction. Based on previous experiences and publications, type A to C lesions were classified as stable and type D to G lesions as unstable (Fig. 1; [5, 6, 15]). In the case of multiple osteolytic lesions per vertebral body, only the most severe lesion was scored. In 57% of the patients, the planning target volume (PTV) comprised more than one tumor affected vertebral body.

### Treatment

For all patients, RT was planned based on planning CTs and performed over one or several 6 MV photon fields. The PTV included the vertebral body/bodies affected by the metastatic spread and the neighboring ones in the cranial and caudal directions. The most common fractionation scheme was 10 × 3 Gy, followed by 5 × 4 Gy and 20 × 2 Gy (Table 1). The indication for palliative RT was individually determined based on disease stage and the patients’ performance status. None of the study patients received additional surgery or other invasive procedures. Further information concerning additional nonsurgical treatments is provided in Table 1.

**Table 1:** Patients’ and treatment characteristics

Characteristics	Value	Percent
*Age (years)*		
Median	75.3	–
Range	70.1–88.4	–
*Gender (n)*		
Female	141	43.8
Male	181	56.2
*Karnofsky PS (%)*		
90	18	5.6
80	82	25.5
70	115	35.7
60	78	24.2
50	21	6.5
40	7	2.2
30	1	0.3
*Number of spinal bone metastases (n)*		
Median	2	–
Range	1–13	–
Solitary	140	43.5
Multiple	182	56.5
*Spine involvement (n)*		
Thoracic	199	61.8
Lumbar	123	38.2
*Primary tumor (n)*		
Breast carcinoma	47	14.6
NSCLC	117	36.3
SCLC	11	3.4
Renal carcinoma	53	16.5
Colorectal cancer	33	10.2
Prostate cancer	13	4.0
Urothelium carcinoma	21	6.5
HCC	2	0.6
ACC (head and neck)	2	0.6
HNSCC	3	0.9
Vulvar carcinoma	2	0.6
Ovarian cancer	2	0.6
Uterine cancer	2	0.6
Malignant melanoma	14	4.3
*Distant extraskeletal metastases (n)*	120	37.3
Brain	30	9.3
Lung	67	20.8
Liver	63	19.6
Skin	18	5.6
*Single radiation dose (Gy)*		
Median	3	–
Range	2–4	–
*Cumulative dose (Gy)*		
Median	30	–
Range	8–40	–
*Fractionation of RT (n)*		
1 × 8 Gy	1	0.3
5 × 4 Gy	3	0.9
10 × 2 Gy	3	0.9
10 × 3 Gy	230	71.4
12 × 3 Gy	1	0.3
14 × 2.5 Gy	27	8.4
20 × 2 Gy	57	17.7
*Indications for RT (n)*		
Pain	272	84.5
Instability	209	64.9
Neurologic deficit	8	2.5
*Chemotherapy (n)*	144	44.7
*Other treatments for bone metastases (n)*		
Orthopedic corset	149	46.3
Bisphosphonates	186	57.8

*Karnofsky PS* Karnofsky performance score;* n* number;* RT* radiotherapy;* Gy* Gray;* NSCLC* non-small cell cancer;* SCLC* small cell cancer;* HCC* hepatic cell cancer;* ACC* adenoid cystic carcinoma;* HNSCC* head and neck squamous cell cancer

### Statistical analysis

Overall survival was defined as the period from start of RT until death from any cause and estimated with the Kaplan–Meier method. Multivariate Cox regression analysis was performed to evaluate possible predictors of overall survival. The distribution of the Taneichi score over time was determined using Bowker’s test; McNemar test was used to assess the distribution of a stable vs. unstable Taneichi score. Univariate ordinal logistic regression analysis was conducted to detect factors associated with the Taneichi score at 3 and 6 months after radiation treatment. Statistical analysis was done using R software, version 3.5.1 (R Core Team 2018, Vienna, Austria). *P*-values < 0.05 were considered statistically significant. The Institutional Review Boards at Heidelberg and Mainz Medical Centers approved this study (S-513/2012).

## Results

The median survival time of the study population after palliative RT of SBM was 5.4 months (95% confidence interval [CI] 4.4–7.2 months). In total, 322 patients presented with a total of 935 osteolytic SBM (range, 1–13 metastases per patient). The baseline characteristics of the study patients are summarized in Table 1.

### Stability analysis

Most patients exhibited unstable SBM prior to RT according to the Taneichi score (i.e., subtypes D–G; 57%, *n* = 183), and 75% of those patients initially presented with associated pain symptoms (*n* = 138). During the follow-up period, RT stabilized primary unstable bone lesions in 13% (*n* = 23/183) and 17% (*n* = 31/183) of the patients as assessed at the first and second follow-up, respectively (*p* < 0.001 for each follow-up, McNemar’s test). When referring only to the patients with unstable SBM still alive at follow-up 1 and 2, the corresponding stabilization rates were 19% (23/118) and 40% (31/78), respectively. Only 1 out of 139 patients with initially stable SBM was observed with a deterioration of stability and was classified unstable in both follow-up examinations. The Bowker test showed significant departure from symmetry in the joint distribution pattern of the scored Taneichi subtypes prior to irradiation and at both follow-up examinations (Tables 2 and 3) (*p* < 0.001 at both follow-ups). At 6 months after palliative RT, highest stabilization rates of primary unstable osteolytic SBM were seen in breast cancer patients (Table 4). In contrast, patients with osteolytic SBM from poorly represented tumor entities in our study population such as malignant melanoma, hepatic cell cancer (HCC), head and neck cancers, gynecologic and genitourinary malignancies mainly exhibited a very poor outcome in terms of stabilization (Table 4). Univariate ordinal logistic regression at follow-up 1 and 2 revealed that several factors correlated with a significant negative stabilization probability of initially unstable SBM after palliative RT compared to breast cancer histologies, among them the presence of pathological fractures before the initiation of RT, lung cancer histology or histology of the group of poorly represented tumors (*summarized in* Table 5). The chance for reaching stabilization and re-ossification in patients with unstable SBM and a KPS below 70% was relatively poor (5%; 3/65) compared to those patients with a KPS of 70% and above (24%; 28/118). Pathological fractures were commonly diagnosed prior to RT (21%; *n* = 68), while post-RT fractures including increasing sintering of previously collapsed vertebrae were evident in only 5% of patients (*n* = 17). In 94% of those post-RT fractures (16/17), SBM were initially rated unstable.

**Table 2:** Test of symmetry for Taneichi score (1st follow-up)

Subtypes at follow-up 1									
		A	B	C	D	E	F	G	Total
*Subtypes before RT*	*A*	*24*	1	0	0	0	0	0	25
	*B*	3	*23*	2	0	0	0	0	28
	*C*	0	2	*34*	1	0	0	0	37
	*D*	2	1	6	*18*	0	0	0	27
	*E*	3	0	4	1	*42*	2	0	52
	*F*	0	1	4	1	1	*27*	0	34
	*G*	2	0	0	0	0	2	*1*	5
	*Total*	34	28	50	21	43	31	1	208

This Bowker test shows the distribution of subtypes of Taneichi score prior to RT and at the first follow-up examination after RT. The evaluation of the distribution of subtypes A to G shows in 16% of the study patients (*n* = 33) an improvement of stability over the course of time. Deterioration of stability occurs only in 3% of the patients (*n* = 6), while in the majority of patients who were still alive at the first follow-up examination (81%, *n* = 169) no change of the stability is evident

*RT* radiotherapy

**Table 3:** Test of symmetry for Taneichi score (2nd follow-up)

Subtypes at follow-up 2									
		A	B	C	D	E	F	G	Total
*Subtypes before RT*	*A*	*18*	1	0	0	0	0	0	19
	*B*	5	*17*	2	0	0	0	0	24
	*C*	4	5	*22*	1	0	0	0	32
	*D*	6	0	7	*7*	0	0	0	20
	*E*	4	2	5	0	*19*	2	0	32
	*F*	0	1	4	0	0	*16*	0	21
	*G*	2	0	0	0	0	2	*1*	5
	*Total*	39	26	40	8	19	20	1	152

This Bowker test shows the distribution of subtypes of Taneichi score prior to RT and at the second follow-up examination after RT. The evaluation of the distribution of subtypes A to G shows in 31% of the study patients (*n* = 47) an improvement of stability over the course of time. Deterioration of stability occurs only in 4% of the patients (*n* = 6), while in the majority of patients who were still alive at the second follow-up examination (65%, *n* = 99) no change of the stability is evident

*RT* radiotherapy

**Table 4:** Tumor entity specific stabilization rates of primary unstable osteolytic SBM

Tumor entity	Stabilization rate at FU1	Stabilization rate at FU2
*Breast cancer*	27% (8/30)	53% (10/19)
*Lung cancer*	21% (12/57)	42% (16/38)
NSCLC	23% (12/53)	42% (16/38)
SCLC	0% (0/4)	NA
*Renal cancer*	22% (2/9)	29% (2/7)
*Colorectal cancer*	14% (1/7)	40% (2/5)
*HCC*	0% (0/2)	0% (0/1)
*HNSCC*	0% (0/1)	NA
*ACC*	0% (0/1)	NA
*Malignant melanoma*	0% (0/2)	0% (0/2)
*Uterine cancer*	0% (0/1)	100% (1/1)
*Vulvar cancer*	0% (0/1)	NA
*Prostate cancer*	0% (0/2)	0% (0/1)
*Urothelial cancer*	0% (0/5)	0% (0/4)

*SBM* Spinal Bone Metastases; *FU* Follow-up; *NA* not analyzable (i.e., patient had died), *NSCLC* non-small cell cancer; *SCLC* small cell cancer; *HCC* hepatic cell cancer; *ACC* adenoid cystic carcinoma; *HNSCC* head and neck squamous cell cancer

**Table 5:** Analysis of prognostic factors related to stabilization of initially unstable SBM

	*1st follow-up*			*2nd follow-up*		
Predictor	*p*-value	OR	CI	*p*-value	OR	CI
Age	0.243	1.05	0.968–1.140	0.224	1.05	0.970–1.140
Poorly represented cancers^a^ (vs. breast cancer)	0.016	4.29	1.309–14.090	0.021	3.47	1.209–9.970
Colorectal cancer (vs. breast cancer)	0.388	1.92	0.435–8.520	0.815	0.86	0.244–3.030
Renal cancer (vs. breast cancer)	0.476	1.69	0.398–7.210	0.167	2.90	0.640–13.130
Lung cancer (vs. breast cancer)	0.048	2.33	1.009–5.370	0.029	2.48	1.096–5.630
KPS (<70% vs. ≥70%)	0.551	1.01	0.977–1.040	0.464	0.99	0.956–1.020
Chemotherapy (yes vs. no)	0.723	1.13	0.567–2.270	0.821	0.93	0.483–1.780
Location (thoracic vs. lumbar spine)	0.526	1.26	0.613–2.600	0.829	1.08	0.555–2.080
Number of SBM (1 vs. >1)	0.906	1.01	0.880–1.160	0.426	1.06	0.921–1.210
Extraskeletal metastases (yes vs. no)	0.206	1.67	0.755–3.700	0.648	1.18	0.582–2.390
Bisphosphonates (yes vs. no)	0.662	1.18	0.556–2.520	0.182	1.63	0.797–3.310
Fractures before RT (yes vs. no)	0.037	2.81	1.066–7.40	0.026	2.72	1.127–6.58

*KPS* Karnofsky performance score,* OR* Odds Ratio, *CI* Confidence limits of the results for a confidence level of 95%,* SBM* Spinal bone metastases,* RT* Radiotherapy

^a^ Included SBM of following tumor entities: malignant melanoma, adenoid cystic carcinoma (ACC), hepatic cell cancer (HCC), head and neck squamous cell cancer (HNSCC), ovarian cancer, uterine cancer, vulvar cancer, urothelial cancer, prostate cancer

### Survival analysis

For the complete patient cohort, relatively poor survival was evident after palliative RT. Overall survival rates 3, 6 and 12 months after RT amounted to 66% (95% CI 61–72%), 48% (95% CI 43–54%) and 29% (95% CI 24–34%); the median survival following RT was 5.4 months (95% CI 4.4–7.2 months).

**Fig. 2:**
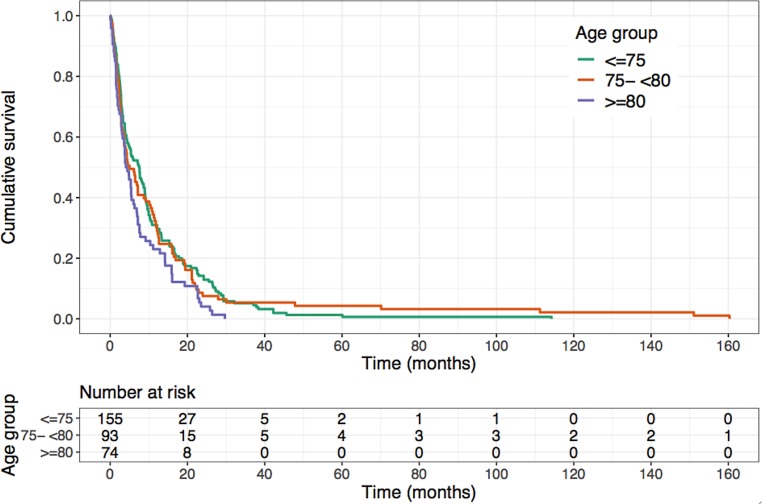
Kaplan–Meier estimation of overall survival depending on the patients’ age showing a strong trend towards a worse overall survival for patients aged at least 80 years (*p* = 0.06, log-rank test)

To analyze the association of age with overall survival, we stratified the study patients into 3 age groups (i.e., 70–74 years, 75–79 years and ≥80 years) and found a strong tendency towards worse survival after RT for patients ≥80 years, albeit not reaching statistical significance (*p* = 0.06; Fig. 2). The corresponding median overall survival was 7.6 months (95% CI 4.8–9.0 months) for patients aged 70–74 years, 5.0 months (95% CI 3.6–9.2 months) for patients aged 75–79 years and 4.3 months (95% CI 3.2–6.3 months) for patients aged 80 years or older.

**Fig. 3:**
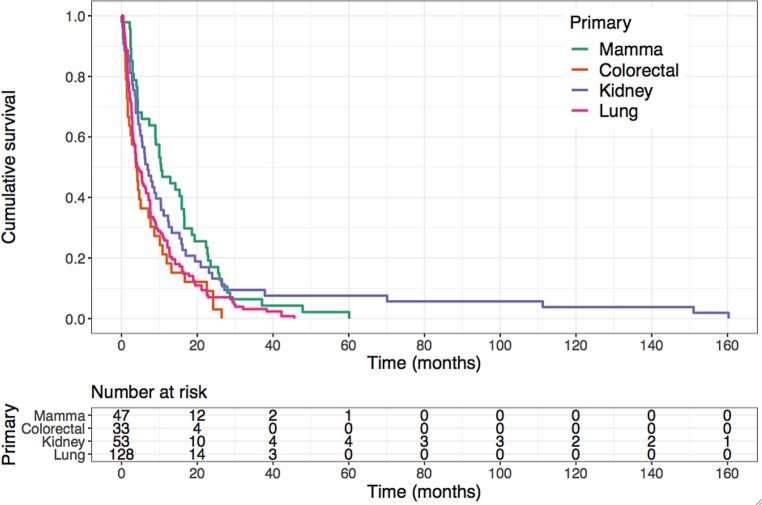
Kaplan–Meier estimation of overall survival depending on the histology of the primary tumor showing a significantly reduced prognosis for lung and colorectal cancer patients compared to breast cancer patients (*p* = 0.003, log-rank test)

In comparing overall survival for patients with the 4 most common primary tumor histologies (i.e., breast, renal, colorectal and lung cancer) accounting for 81% of the study population (261/322 patients), we found significantly better survival rates for breast cancer patients and a significantly worse prognosis for lung and colorectal cancer patients (*p* = 0.003; Fig. 3).

**Fig. 4:**
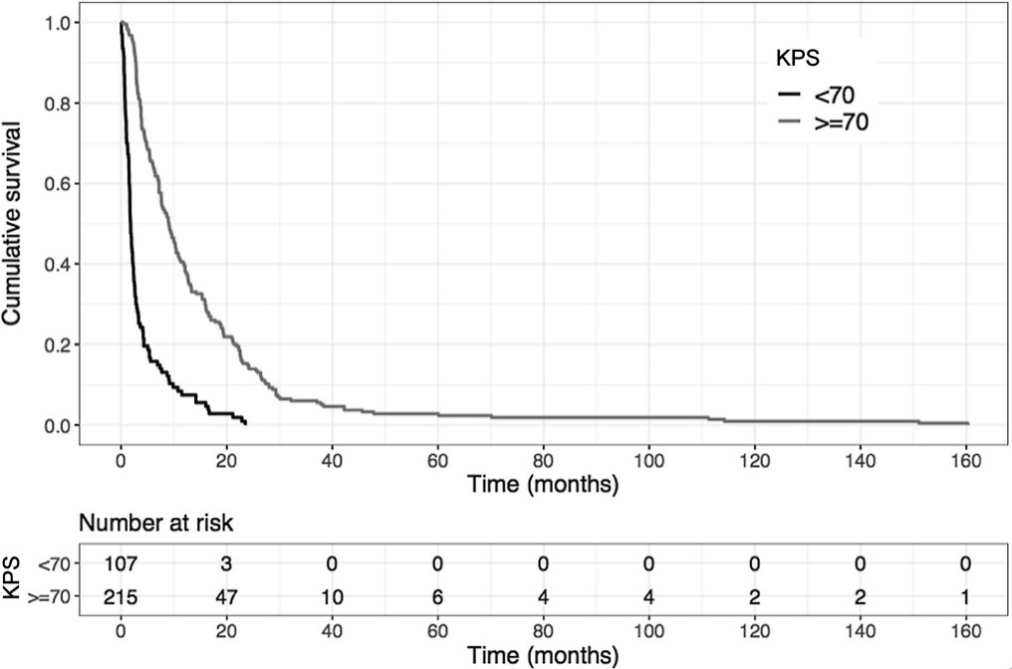
Kaplan–Meier estimation of overall survival depending on the performance score showing a significantly improved prognosis for patients with a Karnofsky performance score (KPS) of at least 70% compared to patients with a KPS <70% (*p* < 0.001, log-rank test)

**Fig. 5:**
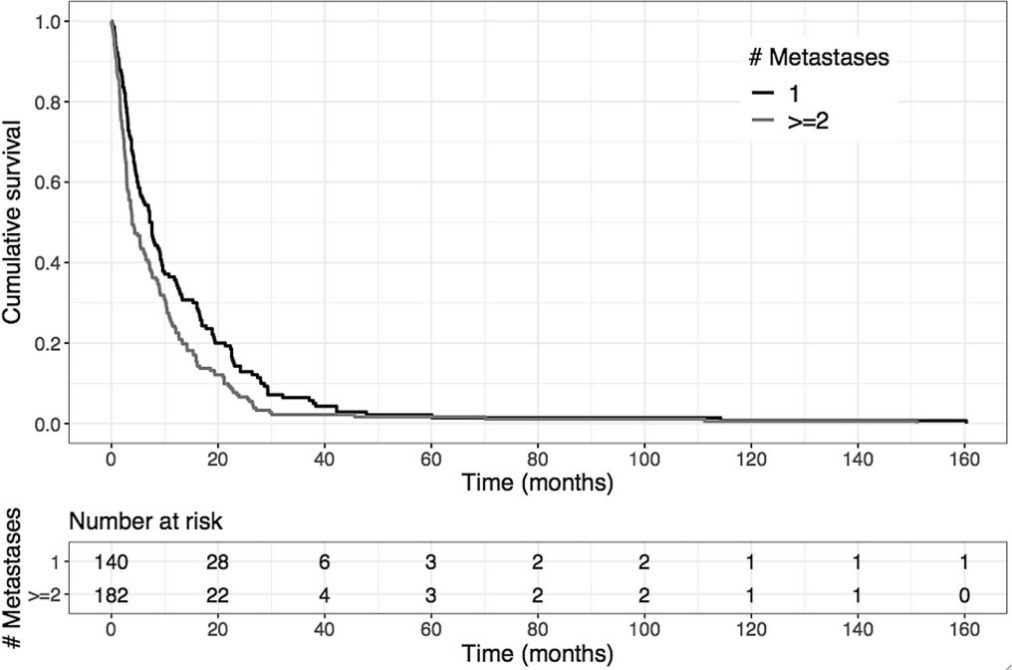
Kaplan–Meier estimation of overall survival depending on the number of spinal bone metastases (SBM) showing a significantly improved prognosis for patients with only 1 SBM compared to patients with at least 2 SBM (*p* = 0.029, log-rank test)

Using multivariate analyses, KPS was found to be the strongest predictive factor for overall survival after RT (*p* < 0.001, multivariate Cox regression analysis; Table 6). In patients with KPS <70%, median overall survival was only 1.8 months (95% CI 1.6–2.3 months) compared to 9.1 months (95% CI 7.6–10.5 months) for patients with KPS ≥70% (*p* < 0.001; Fig. 4). Furthermore, the number of SBM, the application of chemotherapy and a tumor histology from the pooled group of poorly represented entities were significant prognostic factors in the multivariate analysis (Table 6)*. *In patients with only 1 SBM, median overall survival reached 7.4 months (95% CI 5.4–9.2 months) compared to 3.9 months (95% CI 3.3–6.3 months) in those patients with more than 1 SBM (*p* = 0.029; Fig. 5). Administration of chemotherapy extended median overall survival from 4.9 months (95% CI 3.9–7.2 months) to 5.8 months (95% CI 4.4–8.7 months; *p* = 0.046).

**Table 6:** Analysis of prognostic factors related to overall survival after palliative RT

	*Univariate testing*			*Multivariate testing*		
Predictor	*p*-value	HR	CI	*p*-value	HR	CI
Age	0.197	1.017	0.991–1.043	0.507	1.009	0.982–1.038
Poorly represented cancers^a^ (vs. breast cancer)	0.029	1.529	1.044–2.240	0.016	1.663	1.103–2.509
Colorectal cancer (vs. breast cancer)	0.007	1.858	1.188–2.907	NA	1.548	0.879–2.725
Renal cancer (vs. breast cancer)	0.737	1.071	0.718–1.598	NA	1.116	0.665–1.872
Lung cancer (vs. breast cancer)	0.008	1.576	1.127–2.206	NA	1.602	1.133–2.265
KPS (<70% vs. ≥70%)	<0.001	0.954	0.944–0.964	<0.001	0.953	0.942–0.964
Chemotherapy (yes vs. no)	0.151	1.177	0.943–1.469	0.036	1.295	1.018–1.647
Location (thoracic vs. lumbar spine)	0.523	1.077	0.858–1.351	0.664	1.054	0.832–1.335
Number of SBM (1 vs. >1)	0.004	1.066	1.021–1.112	0.031	1.055	1.006–1.107
Extraskeletal metastases (yes vs. no)	0.276	1.134	0.904–1.423	0.711	0.953	0.740–1.228
Bisphosphonates (yes vs. no)	0.381	0.905	0.724–1.131	0.295	0.834	0.596–1.167
Fractures before RT (yes vs. no)	0.098	1.258	0.958–1.651	0.383	1.138	0.853–1.518

*KPS* Karnofsky performance score*, HR* Hazard Ratio*, CI* Confidence limits of the results for a confidence level of 95%, *SBM* Spinal bone metastases, *RT* Radiotherapy, *NA* not analyzable

^a^ Included SBM of following tumor entities: Malignant melanoma, adenoid cystic carcinoma (ACC), hepatic cell cancer (HCC), head and neck squamous cell cancer (HNSCC), ovarian cancer, uterine cancer, vulvar cancer, urothelial cancer, prostate cancer

In the univariate analysis, the only further predictive factor associated with a poor overall survival was tumor histology, with lung and colorectal cancer histologies correlating with a reduced survival (Table 6). Patients’ age, the prevalence of additional extraskeletal metastases, the location of SBM, the concurrent administration of bisphosphonates and the presence of pathological fractures prior to RT were not statistically significant predictors of overall survival (Table 6).

## Discussion

While the analgesic effect of RT on bone metastases has already been well investigated, there is a lack of study data concerning the stabilizing effect of RT on unstable osteolytic metastases [12]. In recent years, we and other groups focused on assessment of the remineralization process of osteolytic SBM after RT [16]. As a result, we found substantial differences of recalcification and stabilization rates of initially unstable SBM depending on the histology [5, 6, 15, 17–19]. Also in this dataset, tumor histology was found strongly associated with differing stabilization rates after palliative RT, and stabilization rates may correspond to individual radiation sensitivities of various tumor cell types, although the exact underlying mechanism regulating radiation-induced recalcification of osteolytic skeletal metastases remains to be elucidated. Recalcification may also depend on the individual tumor micromilieu and the influence of concurrent systemic treatments. In this context, the patients’ age may be an important factor for recalcification of unstable vertebral metastases of various solid tumors. Elderly patients exhibit an increased level of comorbidities that have the potential to influence bone stability and recalcification in the context of bone metastases, among them diabetes mellitus, renal insufficiencies or osteoporosis. Several publications have indicated an influence of bone mineral density and especially osteoporosis on the stability of bone metastases, although no clear evidence is available regarding the stabilization rates following palliative RT in these patients [20, 21]. To the best of our knowledge, no scientific data concerning the stabilization rates have been reported from an explicitly elderly patient cohort, a primary impetus for conducting this retrospective pooled analysis.

Prior to irradiation, 57% of our study patients exhibited unstable SBM in the thoracic or lumbar spinal column. Within 3 and 6 months after palliative RT, significant recalcification and stabilization were observed in up to 40% of the analyzed patients still alive at the time of follow-up; a deterioration of stability was measured in only one patient during the follow-up period. The highest stabilization rates in our study population were found in elderly breast cancer patients, while elderly patients with SBM from lung cancer, colorectal cancer, renal cancer or further poorly represented tumor entities such as malignant melanoma, prostate and urothelial cancers showed minor or negligible recalcification following palliative RT. These findings are largely in line with previous published data reporting histology-dependent recalcification in younger patients [6, 15, 18, 19, 22]. Regarding the observed minor stabilization rates in elderly patients with these histologies, alternate approaches for improving the stability of the metastatic vertebral column may need to be considered. Stereotactic ablative radiotherapy (SBRT) may hold the potential to increase the stabilization rates in unstable SBM, particularly for geriatric patients with a good performance score and SBM from tumor entities with a relatively low chance of bone stability after 6 months. However, evidence remains to be provided regarding the effectiveness of SBRT for the stabilization of vertebral metastases in elderly patients. Also, surgical stabilization options may become more relevant for the respective patients; however surgery may interrupt necessary systemic treatments for a considerable time compared to SBRT. Additionally, perioperative morbidity and mortality is likely increased in affected geriatric patients.

When interpreting the stabilization rates of our study, it is important to consider the histology-dependent differing prognoses of patients with bone metastases, as significant recalcification after RT requires at least 3 to 6 months. Thus, the relevance of stabilization of unstable SBM is higher for patients with an average survival exceeding 6 months. In our dataset, patients aged 80 years or older showed a strong trend towards reduced survival rates compared to elderly patients below 80 years (i.e., 39% vs. 51% at 6 months after RT; *p* = 0.06). This may not only be due to the patients’ oncologic outcome, but may also reflect the influence of other age-prevalent causes such as cardiovascular diseases. As reported by many studies before, we also found that the performance status was a strong prognostic factor for predicting the residual life expectancy after palliative RT of SBM in elderly patients [23–27]. Here, patients with a KPS below 70% had an extremely poor overall survival of less than 2 months compared to 9 months for those patients with a KPS of at least 70%. Based on these results, single fraction RT should be the preferred treatment option for elderly patients with a poor general condition and advanced tumor disease, as it has shown equivalent pain control rates compared to more protracted RT schedules [12, 28].

Moreover, our analysis showed that patients with a solitary/singular SBM exhibited an almost doubled median overall survival as compared to patients with 2 or more SBM (7.4 vs. 3.9 months; *p* = 0.031), while the additional survival benefit by administration of chemotherapy was less than 1 month in these elderly patients (5.8 vs. 4.9 months at the median; *p* = 0.036). The effect of oligometastatic cancers and consecutive ablative treatments in elderly metastatic patients has not yet been elucidated and will likely be subject to further research in the future.

Our study has some limitations, especially the retrospective character of this patient cohort. For instance, comorbidities (including age- and cancer therapy-related osteoporosis), the patients’ smoking status, medications and lifestyle factors such as physical exercises could not be routinely assessed in our elderly patient cohort, although it is conceivable that these factors might have an impact on the stabilization and fracture rates after palliative RT as well as overall survival. Second, the used Taneichi score is restricted to score the stability of osteolytic thoracic and lumbar metastases. As a consequence, prostate cancer patients are considerably underrepresented in this elderly cohort, as the majority of SBM from prostate cancer has an osteoplastic phenotype. Since the majority of SBM spread to the thoracic and lumbar spine, we believe that the non-consideration of the cervical and sacral spine represents only a minor limitation. A third limitation is due to the long inclusion time period, many patients in our study were treated before modern systemic therapies such as targeted therapies and checkpoint inhibitors became routinely used in the clinic. While these agents substantially contributed to improved survival times in many metastatic tumor diseases in recent years, their routine use in geriatric patients remains subject to some debate [29–33]. On the other hand, the stabilization effect of palliative radiotherapy in unstable SBM might become better than reported in this study with improved overall survival rates. Nevertheless, results from this large multicenter cohort of patients with an advanced age help to generate hypotheses that may direct further assessment of palliative radiation effects through prospective clinical trials in elderly patients, and these trials are urgently required to address the palliative needs of an increasingly aging population.

## Conclusion

The treatment of elderly patients with unstable SBM is complex and requires a multidisciplinary approach whereby palliative RT has a pivotal role in the treatment concept. Histology and performance status seem to dominate prognosis of elderly patients with SBM and should be considered to determine radiation fractionation in order to reduce hospitalization times in the remaining lifespan.
